# Aortic stiffening precedes onset of heart failure with preserved ejection fraction in patients with asymptomatic diastolic dysfunction

**DOI:** 10.1186/s12872-017-0490-9

**Published:** 2017-02-14

**Authors:** Ilya Karagodin, Omer Aba-Omer, Rodney Sparapani, Jennifer L. Strande

**Affiliations:** 1 Department of Medicine, Medical College of Wisconsin, 9200 Wisconsin Ave, Milwaukee, WI 53226 USA; 2 Division of Biostatistics, Medical College of Wisconsin, Milwaukee, WI USA; 3 Division of Cardiovascular Medicine, Medical College of Wisconsin, Milwaukee, WI USA

**Keywords:** Heart Failure, Heart Failure with Preserved Ejection Fraction (HFpEF), Diabetes, Echocardiography, Cardiomyopathy, Biomarker

## Abstract

**Background:**

Identifying which patients with diastolic dysfunction will progress to heart failure with preserved ejection fraction (HFpEF) remains challenging. The goal of this study is to determine whether increased vascular stiffness as identified on 2D transthoracic echocardiography (TTE) serves as a biomarker for the development of HFpEF in patients with diastolic dysfunction.

**Methods:**

The study design is a matched retrospective case–control study. Subjects with diastolic dysfunction were divided into two groups based on whether they had a clinical diagnosis of HFpEF. The two groups were matched based on age, gender, race and body surface area, resulting in 77 matched pairs (*n* = 154). Data from the first TTE that documented diastolic dysfunction prior to the development of HFpEF was extracted along with baseline demographic and clinical data. Indices of vascular stiffness were measured and compared. A sub-group analysis was performed to compare diabetic subjects in Group 1 (*n* = 43) to those in Group 2 (*n* = 21).

**Results:**

Group 1 had significantly decreased aortic distensibility as measured on the initial TTE when compared to Group 2 (1.9 ± 1.0 vs. 2.8 ± 1.8 cm^2^dyne^−1^10^−3^, *p* = 0.01). In the diabetic subset, Group 1 had significantly less aortic strain (6.9 ± 3.3 vs. 9.7 ± 5.6%, *p* = 0.02) and aortic distensibility (1.8 ± 1.0 vs. 3.5 ± 2.6 cm^2^dyne^−1^10^−3^, *p* = 0.02) compared to Group 2. Other indices of vascular stiffness did not differ significantly between groups.

**Conclusions:**

This study demonstrates that increased proximal aortic stiffness is associated with the development of HFpEF in patients with asymptomatic diastolic dysfunction. Larger prospective studies are needed to further investigate this relationship.

## Background

Diastolic dysfunction is known to be an important contributor to the development of heart failure with preserved ejection fraction (HFpEF) [1]. The pathophysiologic mechanisms that contribute to the continuum between diastolic dysfunction and HFpEF have yet to be fully elucidated. Patients with HFpEF have been shown to have arterial stiffening beyond that associated with normal aging and hypertension [2]. The recoil of the ascending aorta during each cardiac cycle may facilitate early diastolic left ventricular filling [3]. Aortic stiffening, as indicated by decreased aortic distensibility, has been associated with more severe symptoms of heart failure in patients with HFpEF [4]. We therefore hypothesize that increased vascular stiffness in the setting of diastolic dysfunction is associated with the development of HFpEF.

Hypertension, coronary artery disease (CAD), obesity, atrial fibrillation (AF), chronic kidney disease (CKD) and diabetes mellitus (DM) have all been shown to be associated with HFpEF [5–8]. A recent systematic meta-analysis of 27 studies found a significant correlation between arterial stiffness and diastolic dysfunction. Even though it has been speculated that diastolic dysfunction and arterial stiffness may be an important mechanism in the development of HFpEF in these patient populations [9], there has been no study to date that has correlated arterial or aortic stiffness and diastolic dysfunction with the development of HFpEF. DM in particular has been shown to be an independent predictor of morbidity and mortality in patients with heart failure, with the relative risk of cardiovascular death or heart failure hospitalization conferred by DM greater in patients with HFpEF compared to heart failure with reduced ejection fraction [10]. One hypothesis is that increased advanced glycation end product deposition and collagen cross-linking in the diabetic myocardium leads to endothelial dysfunction and increased vascular stiffness, thereby increasing cardiac afterload and myocardial oxygen requirements, ultimately leading to diastolic dysfunction and the subsequent development of HFpEF [11].

Pulse-wave velocity, as measured by applanation tonometry, remains the gold-standard non-invasive method for measuring vascular stiffness. Velocity-encoded magnetic resonance imaging (MRI) has also been shown to have excellent correlation with invasive hemodynamic measurements of aortic stiffness [12]. In addition, non-invasive measurement of aortic distensibility using TTE has been shown to have a high degree of accuracy when compared with invasive measurements in different populations [12, 13]. TTE is widely available at most medical centers and measurements of vascular stiffness can be performed from a routine comprehensive TTE without the need to follow special protocols or obtain additional images, making it an attractive alternative approach to evaluating aortic stiffness in subjects at risk for developing HFpEF.

The goal of this study is to determine whether increased vascular stiffness serves as a biomarker for the subsequent development of HFpEF in patients with diastolic dysfunction and whether this can be identified on TTE. We hypothesize that those patients who progress from asymptomatic diastolic dysfunction to HFpEF, both diabetic and non-diabetic, have a greater degree of vascular stiffness at baseline compared to those that remain asymptomatic.

## Methods

### Study design and patient selection

The study design is a matched case–control study in which data was retrospectively reviewed and collected. The study protocol was approved by the Medical College of Wisconsin Institutional Review Board. Human subject research data was de-identified and stored electronically on a secure, password-protected computer server (REDCap). REDCap servers are securely housed in an on-site limited access data center managed by the Medical College of Wisconsin. All web-based information transmission is encrypted. The data is stored on a private, firewall-protected network. All users are given individual user IDs and passwords and their access is restricted on a role-specific basis. REDCap was developed specifically around HIPAA-security guidelines and is implemented and maintained per Medical College of Wisconsin guidelines.

The echocardiogram database at Froedtert Memorial Lutheran Hospital (FMLH) was used to screen TTEs between 7/1/2003 and 7/1/2013. The search terms included (1) diastolic dysfunction and (2) preserved ejection fraction (EF > 50%) and excluded (1) systolic dysfunction, (2) normal diastolic function, (3) E/A fusion, (4) mitral valve abnormalities, (5) severe aortic stenosis, (6) severe mitral regurgitation, (7) annuloplasty and/or (8) bioprosthetic valves. After accounting for serial studies, 561 subjects qualified for the study. Subjects were further excluded if they had missing clinical data (*n* = 63) in the EHR, any serial TTE report that included an EF of <50% (*n* = 40), non-diagnostic echocardiograms (*n* = 10), or if they had previously undergone heart transplantation (*n* = 1).

**Fig. 1:**
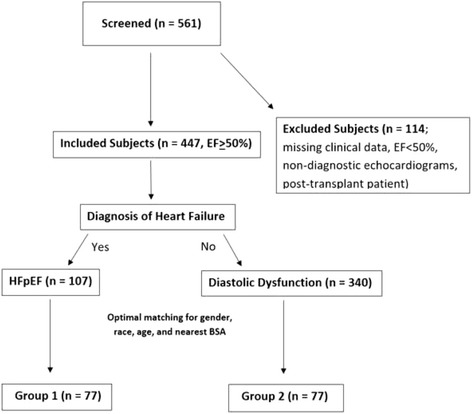
Flow diagram of study. Flow diagram illustrating method of subject selection, including exclusion and matching criteria

Among the remaining subjects (*n* = 447), the electronic health record (EHR) was reviewed to identify those subjects who had heart failure with preserved ejection fraction. Subjects were assigned to Group 1 (*n* = 107) if their EHR contained an ICD-9 diagnosis of congestive heart failure and clinical documentation of at least one of the following signs or symptoms of heart failure by the end of the study period: shortness of breath, weight gain, orthopnea, paroxysmal nocturnal dyspnea or increased leg swelling. Subjects were placed into Group 2 (*n* = 340) if they remained free of heart failure throughout the study period. After information on gender, race, age and body surface area (BSA) was collected, the subjects were optimally matched for these variables to yield 77 matched pairs (*n* = 154) of subjects which were ultimately included in our study (Fig. 1).

For each subject, TTE reports were screened in a retrospective fashion until the earliest study that documented diastolic dysfunction was identified, from which echocardiographic data was extracted. Echocardiographic data was initially extracted from the clinical report which was generated by a board-certified cardiologist. This data included: blood pressure (BP), left ventricular posterior wall thickness at end-diastole (LVPWd) and end-systole (LVPWs), left ventricular (LV) mass, LV mass index, LV ejection fraction (LVEF) as measured by Simpson’s equation, fractional shortening (FS), LV internal diameter at end-diastole (LVIDd) and end-systole (LVIDs), LV end-diastolic volume (LVEDV) and index, LV end-systolic volume (LVESV) and index, relative wall thickness (RWT), left atrial linear dimension, left atrial volume index, mitral peak E velocity, mitral peak A velocity, E/A ratio, stroke volume (SV) and stroke volume index (SVI). Left ventricular volumes (EDV and ESV) were calculated using the biplane method of disks (modified Simpson’s rule). SV, LVEF and FS were determined based on the American Society of Echocardiography/European Association of Cardiovascular Imaging (ASE/EACVI) guidelines [14] and reported in Table 1. Diastolic function was determined and graded by the interpreting cardiologist according to the recommendation of the American Society of Echocardiography 2002 and 2009 guidelines. The 2002 guidelines identified diastolic dysfunction as having a lower E than A-wave velocity with a prolonged isovolumic relaxation time and shortened deceleration time [15]. Mitral annular tissue e’ and a’ velocities were not routinely measured or reported until 2009 when diastolic function grading was further defined as: normal diastolic function: E/A ≥1, average e’ >9 cm/s; mild diastolic dysfunction: E/A < 1, average e’ ≤ 9 cm/s, moderate diastolic dysfunction: E/A ≥ 1, average e’ ≤ 9 cm/s, severe diastolic dysfunction: E/A ≥ 2, average e’ ≤ 9 [16]. Because mitral annular tissue velocities were not included on the reports until 2009, we did not report this data in the study.

**Table 1:** Study calculations [12, 34–37]

Variable	Formula
Pulse Pressure (PP; mmHg)	systolic blood pressure (SBP) – diastolic blood pressure (DBP)
Diastolic Wall Strain (%)	(LVPWs-LVPWd)*100/LVPWs
End Systolic Pressure (mmHg)	0.9*SBP
Arterial Stiffness (mmHg/mL/m^2^)	PP/SVI
Arterial Elastance (mmHg/mL)	ESP/SV
Aortic Strain (%)	(AoS-AoD)*100/AoD
Aortic Distensibility (cm^2^dyne^−1^10^−3^)	2* [(AoS-AoD)/(AoD*PP)] * 1000
Relative Wall Thickness (cm)	2*(LVPWd)/LVIDd
LV Mass (g)	0.8* [1.04 * (IVSd + LVIDd + LVPWd) ^3^ - LVIDd^3^] + 0.6 g
Fractional Shortening (%)	(LVIDd-LVIDs)/LVIDd*100
Stroke Volume (mL)	EDV - ESV
Ejection Fraction (%)	(SV/EDV) * 100

Additional clinical data including hypertension, CAD, DM, AF, CKD (estimated GFR <60 mL/min/1.73 m^2^ for 3 months or more), alcohol and tobacco use, N-terminal pro-hormone brain natriuretic peptide (NT-proBNP) level, glomerular filtration rate (GFR, estimated by the CKD-EPI equation [17]), and data on medication use (beta blockers, calcium channel blockers, ACE inhibitors and angiotensin receptor blockers) was extracted from the EHR at FMLH.

### Assessment of arterial and aortic stiffness

**Fig. 2:**
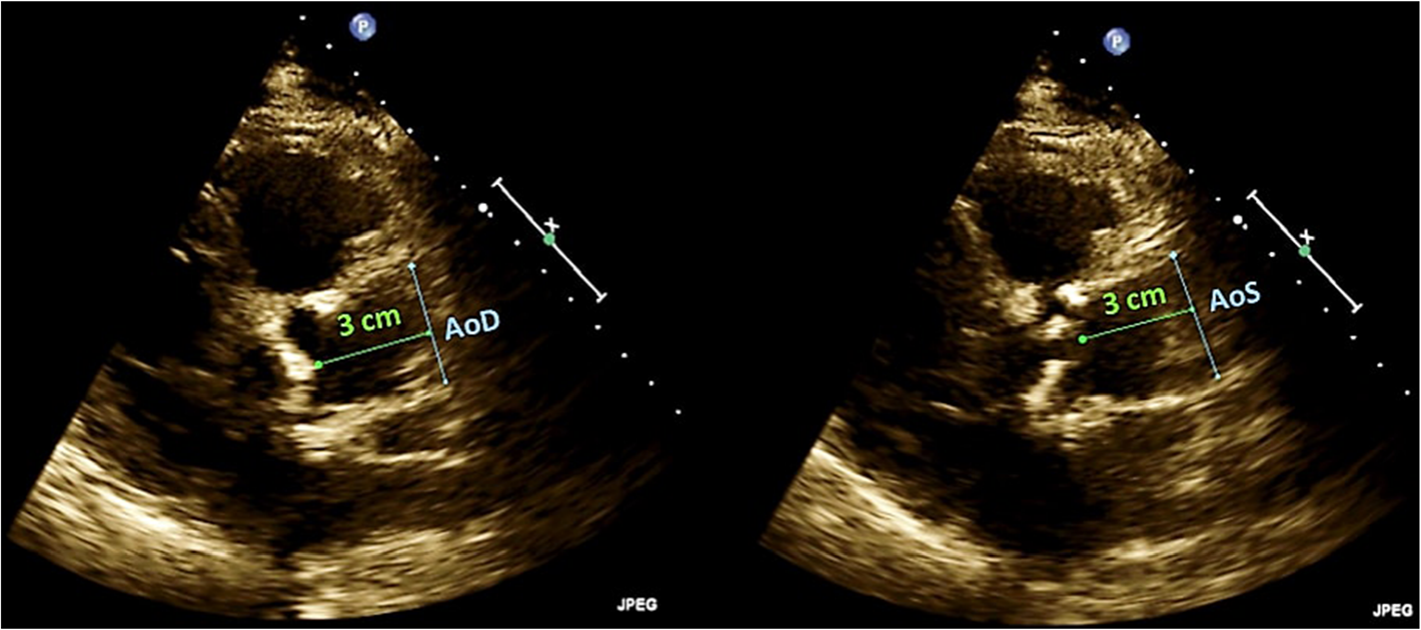
Ascending aortic diameter measurements. Ascending aortic diameters were measured three centimeters above the aortic valve at end-diastole (AoD) and end-systole (AoS) in the 2D parasternal view

From the earliest TTE documenting diastolic dysfunction in study subjects and prior to Group 1 subjects developing heart failure, the dimensions of the ascending aorta were measured. Ascending aortic diameters were measured three centimeters above the aortic valve at end-diastole (AoD) and end-systole (AoS) in the 2D parasternal view (Fig. 2). These measurements were used to calculate aortic distensibility and aortic strain using formulas reported in Table 1. Arterial stiffness and arterial elastance were calculated as reported in Table 1 and included the SV as determined from the same TTE that was used to measure aortic dimensions.

### Additional calculations

The following calculations that were not included in the clinical report were performed, as outlined in Table 1: pulse pressure (PP), diastolic wall strain (DWS), end systolic pressure (ESP), arterial stiffness, arterial elastance, aortic strain, aortic distensibility and relative wall thickness (RWT). Missing blood pressure recordings at the time of the earliest echocardiogram documenting diastolic dysfunction prevented the calculation of some indices of vascular stiffness in 48/77 subjects in Group 1 and 28/77 subjects in Group 2.

For reference, we used previous literature that published mean adult values for aortic distensibility (in cm^2^dyne^−1^10^−3^) and aortic strain (%) in control subjects (10 ± 5.1 and 18 ± 8.0, respectively), hypertensive subjects (3.1 ± 1.5 and 11 ± 7.0, respectively), diabetic subjects (5.1 ± 2.8 and 9 ± 3.0, respectively) and subjects with both hypertension and diabetes (2.0 ± 0.9 and 8 ± 3.0, respectively) [18].

### Statistical analysis

The data analysis was performed using Statistical Analysis System (SAS) software, version 9.3. Continuous variables are expressed in the Tables as mean ± standard deviation and categorical variables as a percentage. For the matched pairs in our study (Group 1 vs. Group 2), McNemar’s statistic was used to compare qualitative variables and a one-sample t-test was used to compare quantitative variables. A *p*-value of <0.0125 was considered statistically significant, based on the Bonferroni correction for multiple comparisons.

We performed a multiple logistic regression analysis to determine whether aortic distensibility is associated with higher predictive risk when adjusting for age, gender, race, BSA, hypertension, DM and CKD. To calculate the optimal cut-off value for aortic distensibility, we fit a simple logistic regression and generated a receiver operating characteristic (ROC) curve to achieve the highest possible level of sensitivity and specificity, and to maximize the area under the curve (AUC).

We also performed simple linear regressions, eliminating outliers more than two standard deviations outside of the mean, to evaluate whether aortic distensibility is correlated to other clinical or echocardiographic parameters, specifically age, gender, race, body surface area, hypertension, DM, CKD, LV mass, LV mass index, LV internal diameter end-diastole, LV-end diastolic volume and index, relative wall thickness and E/A velocity.

An unmatched sub-group analysis was also performed to compare the diabetic patients in each group (Group 1 vs. Group 2). A one-way analysis of variance (ANOVA) was used to compare the differences between groups. A *p*-value of <0.05 was considered statistically significant for the sub-group analysis.

## Results

### Baseline characteristics

The baseline characteristics of the two groups are shown in Table 2. Subjects in Group 1 had significantly more hypertension (*p* = 0.02), DM (*p* = 0.0002), CAD (*p* = 0.01), and AF (*p* = 0.003), and subjects in Group 1 had significantly lower GFRs (*p* = 0.002) and significantly more clinical diagnoses of CKD (p < 0.0001) compared to those in Group 2.

**Table 2:** Baseline characteristics

Variable	Group 1: HFpEF (*n* = 77)	Group 2: Diastolic Dysfunction (*n* = 77)	*P*-value
Age (years)	68.6 ± 9.9	68.5 ± 9.9	0.96
Gender (% Female)	67.5	67.5	1.0
Race (% African American)	30.5	30.5	1.0
Body Surface Area (m^2^)	1.97 ± 0.31	1.93 ± 0.24	0.31
Tobacco Use (%)	56.2	60.3	0.61
Alcohol Use (%)	49.3	58.0	0.27
Hypertension (%)	88.3	72.7	0.02
Diabetes (%)	55.8	27.3	0.0002
Coronary Artery Disease (%)	61.0	39.0	0.01
Atrial Fibrillation (%)	40.3	16.9	0.003
Chronic Kidney Disease (%)	63.6	24.7	<0.0001
NT-proBNP (pg/mL)	9810 ± 14308	2148 ± 3026	0.07
GFR (mL/min/1.73 m^2^)	44.8 ± 20.0	57.9 ± 23.6	0.002
Systolic BP (mmHg)	145.9 ± 29.7	140.9 ± 21.0	0.73
Diastolic BP (mmHg)	70.7 ± 15.7	74.6 ± 10.9	0.37
Pulse Pressure (mmHg)	75.6 ± 21.5	66.3 ± 16.7	0.17
Beta Blockers (%)	62.3	72.7	0.21
Calcium Channel Blockers (%)	28.6	28.6	1.0
ACE Inhibitors (%)	16.9	26.0	0.13
Angiotensin Receptor Blockers (%)	18.2	23.4	0.45

*Note*: Continuous data expressed as mean ± standard deviation. Categorical variables expressed as percentage

In the overall cohort, there were no significant differences between the number of patients on beta blockers, calcium channel blockers, ACE inhibitors or angiotensin receptor blockers in Group 1 versus Group 2 (Table 2).

### Echocardiographic measurements

The echocardiographic measurements of the two groups are shown in Table 3. Group 1 had significantly increased left ventricular posterior wall thickness at end-diastole (*p* = 0.03) and end-systole (*p* = 0.0001), as well as increased left ventricular mass (*p* = 0.0004), left ventricular mass index (*p* = 0.001), left ventricular internal diameter at end-diastole (*p* = 0.006), and left ventricular internal diameter at end-systole (*p* = 0.02) compared to Group 2. Group 1 also had significantly increased left atrial linear dimension (*p* = 0.028) and mitral peak A velocity (*p* = 0.048) compared to group 2. There were no significant differences in diastolic wall strain (*p* = 0.23). No significant differences were observed with respect to other TTE measurements including severity of diastolic dysfunction (Table 3).

**Table 3:** Transthoracic echocardiogram measurements

Measurement	Group 1: HFpEF (*n* = 77)	Group 2: Diastolic Dysfunction (*n* = 77)	*P*-value (matched pairs only)
LV posterior wall thickness end-diastole (cm)	1.20 ± 0.25 - (77)	1.11 ± 0.22 - (77)	0.03
LV posterior wall thickness end-systole (cm)	1.85 ± 0.38 - (75)	1.64 ± 0.26 - (72)	0.0001
LV mass (g)	217.9 ± 72.7 - (72)	177.9 ± 58.5 - (74)	0.0004
LV mass index (g/m^2^)	110.9 ± 34.5 - (44)	91.3 ± 26.8 - (71)	0.001
LVEF – Simpson’s (%)	61.8 ± 6.37 - (59)	60.3 ± 5.52 - (66)	0.25
Fractional shortening index (%)	33.5 ± 9.36 - (76)	34.5 ± 10.3 - (74)	0.48
LV internal diameter end-diastole (cm)	4.67 ± 0.70 - (77)	4.40 ± 0.57 - (76)	0.006
LV internal diameter end-systole (cm)	3.09 ± 0.60 - (77)	2.88 ± 0.61 - (76)	0.02
LV end-diastolic volume (mL)	87.8 ± 34.5 - (63)	84.9 ± 30.4 - (67)	0.99
LV end-diastolic volume index (mL/m^2^)	47.6 ± 16.1 - (21)	57.6 ± 17.3 - (17)	0.90
Diastolic Wall Strain (%)	34.0 ± 12.0 - (75)	32.0 ± 9.0 - (72)	0.23
LV end-systolic volume (mL)	35.3 ± 16.0 - (60)	33.5 ± 12.7 - (68)	0.83
LV end-systolic volume index (ml/m^2^)	18.8 ± 8.45 - (20)	21.8 ± 7.84 - (17)	0.90
Relative wall thickness (cm)	0.57 ± 0.16 - (23)	0.50 ± 0.08 - (16)	0.97
Left atrial linear dimension (cm)	4.10 ± 0.64 - (76)	3.89 ± 0.60 - (77)	0.03
Mitral peak E velocity (m/s)	1.09 ± 0.28 - (74)	1.01 ± 0.26 - (77)	0.052
Mitral peak A velocity (m/s)	0.89 ± 0.32 - (73)	0.79 ± 0.25 - (75)	0.048
E/A ratio	1.37 ± 0.60 - (72)	1.36 ± 0.48 - (73)	0.68
Ascending aortic root diameter end-diastole (cm	3.08 ± 0.39 - (75)	3.04 ± 0.43 - (72)	0.54
Ascending aortic root diameter end-systole (cm)	3.31 ± 0.37 - (75)	3.29 ± 0.42 - (72)	0.86
Stroke volume (mL)	81.4 ± 24.5 - (28)	77.7 ± 17.1 - (38)	0.82
Stroke volume index (mL/m^2^)	41.1 ± 13.3 - (27)	40.4 ± 10.5 - (38)	0.74
LV end systolic pressure (mmHg)	131.4 ± 26.7 - (29)	126.9 ± 18.9 - (49)	0.73
Mild Diastolic Dysfunction (%)	24.7	9.1	0.04^*^
Moderate Diastolic Dysfunction (%)	71.4	83.1	
Severe Diastolic Dysfunction (%)	3.9	7.8	

*Note*: Continuous data expressed as mean ± standard deviation. Categorical variables expressed as percentage. Group size is listed in parentheses

^*^ *p*-value calculated using conditional logistic regression with two degrees of freedom

### Assessment of arterial and aortic stiffness

Group 1 (*n* = 29) had a significant decrease in aortic distensibility compared to Group 2 (*n* = 48; *p* = 0.01). No significant differences were observed with respect to arterial stiffness, arterial elastance and aortic strain (Table 4).

**Table 4:** Markers of vascular stiffness

Variable	Group 1: HFpEF (*n* = 77)	Group 2: Diastolic Dysfunction (*n* = 77)	*P*-value
Arterial Stiffness (mmHg/mL/m^2^)	2.0 ± 0.69 - (22)	1.7 ± 0.52 – (30)	0.49
Arterial Elastance (mmHg/mL)	1.7 ± 0.50 - (22)	1.7 ± 0.40 – (30)	0.80
Aortic Strain (%)	7.5 ± 3.6 – (75)	8.6 ± 4.2 – (72)	0.22
Aortic Distensibility (cm^2^dyne^−1^10^−3^)	1.9 ± 1.0 – (29)	2.8 ± 1.8 – (48)	0.01

*Note*: All data expressed as mean ± standard deviation. Group size is listed in parentheses

To further explore the predictive value of decreased aortic distensibility as an independent risk factor for the development of HFpEF, we performed a multiple logistic regression analysis corrected for age, BSA, gender, race, HTN, DM and CKD. The measure of aortic distensibility was a significant predictor of the development of HFpEF [odds ratio = 0.61 and 95% confidence interval (0.39–0.96)].

**Fig. 3:**
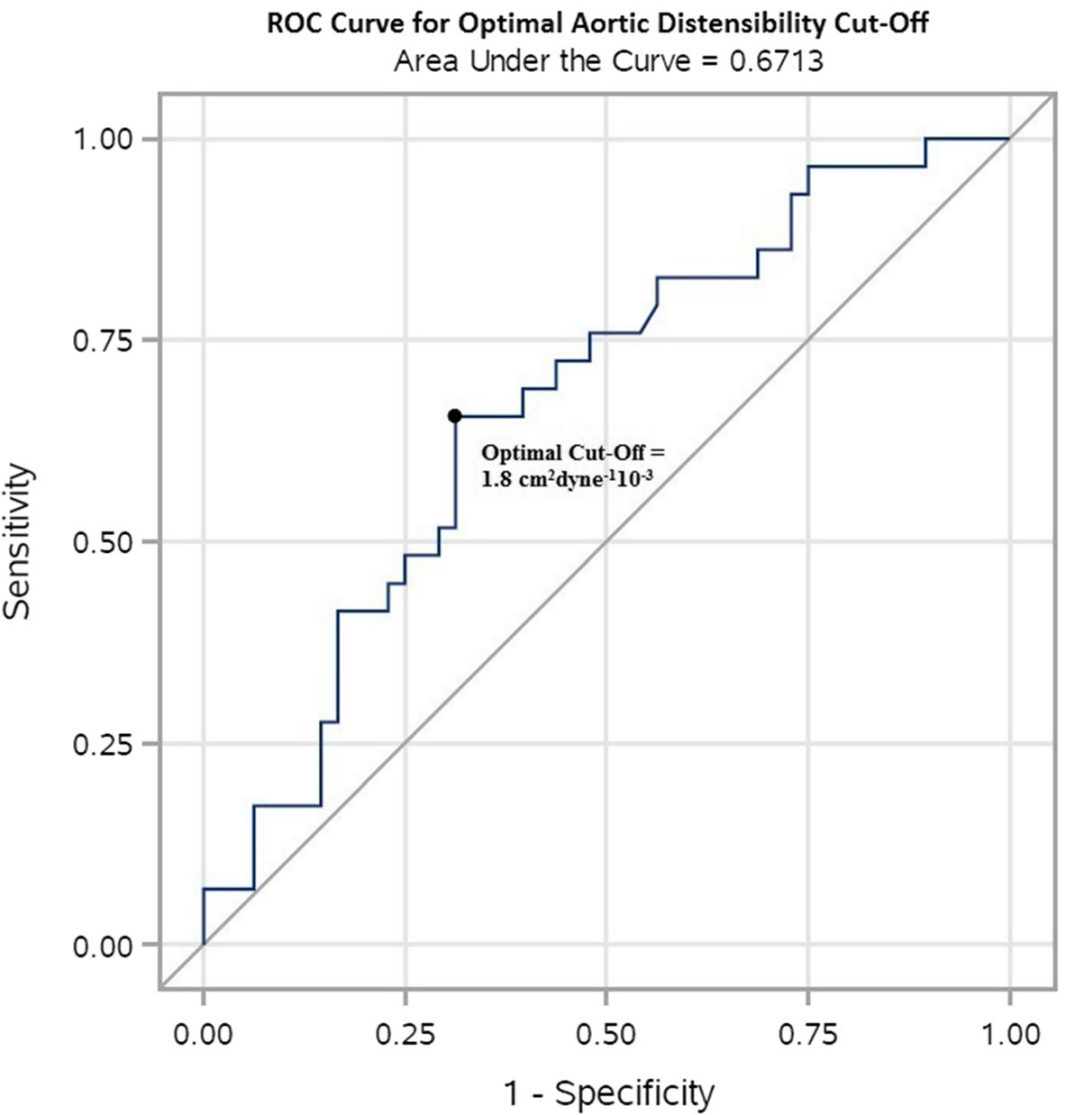
ROC curve for optimal aortic distensibility cut-off. ROC curve showing optimal aortic distensibility cut-off of 1.8 cm^2^dyne^−1^10^−3^, with 62.1% sensitivity and 68.8% specificity. The AUC of the ROC curve is 0.671

ROC curves were generated for aortic distensibility as an indicator of future HFpEF development. If aortic distensibilty was not included in the model but all other independent variables (age, BSA, gender, race, HTN, DM and CKD) remained the same, the area under the ROC curve was 0.796. Adding aortic distensibility to the predictive model boosted the area under the curve to 0.815. Therefore, at the time diastolic dysfunction is first detected by echocardiogram, the addition of aortic distensibilty increases the predictive value of the underlying co-morbidities for HFpEF development. To determine an optimal cutoff for aortic distensibility, we fit a simple logistic regression to arrive at a cutoff of 1.84 cm^2^dyne^−1^10^−3^ with 62.1% sensitivity and 68.8% specificity (Fig. 3).

Furthermore, using simple linear regressions that eliminated outliers more than two standard deviations outside of the mean, aortic distensibility was poorly correlated to age (*r* ^*2*^ = 0.007), gender (*r* ^*2*^ = 0.003), race (*r* ^*2*^ = 0.004), BSA (*r* ^*2*^ = 0.00002), hypertension (*r* ^*2*^ = 0.11), DM (*r* ^*2*^ = 0.02), CKD (*r* ^*2*^ = 0.08), LV mass (*r* ^*2*^ = 0.04), LV mass index (*r* ^*2*^ = 0.05), LV internal diameter end-diastole (*r* ^*2*^ = 0.03), LV end-diastolic volume (*r* ^*2*^ = 0.03), LV end-diastolic volume index (*r* ^*2*^ = 0.04), relative wall thickness (*r* ^*2*^ = 0.03), and E/A velocity (*r* ^*2*^ = 0.07).

### Diabetic sub-group analysis

A sub-group analysis was performed to compare diabetic subjects in Group 1 (*n* = 43) to diabetic subjects in Group 2 (*n* = 21). There were no significant differences observed between groups in terms of age, gender, race, BSA, tobacco use, alcohol use, CAD, hypertension, AF or cardiac medication usage (Table 5). The vascular stiffness measurements, as in the main cohort, were limited by missing blood pressure recordings. Despite this, Group 1 (*n* = 43) was noted to have significantly decreased aortic strain compared to Group 2 (*n* = 9; *p* = 0.02). Aortic distensibility was also significantly decreased in Group 1 (*n* = 17) compared to Group 2 (*n* = 14; *p* = 0.02). Differences in arterial stiffness and arterial elastance did not reach statistical significance (Table 6).

**Table 5:** Baseline characteristics for diabetic subset

Variable	Group 1: Diabetic HFpEF (*n* = 43)	Group 2: Diabetic Diastolic Dysfunction (*n* = 21)	*p*-value
Age (years)	66.0 ± 9.40	66.9 ± 10.2	0.73
Gender (% Female)	65.1	71.4	0.61
Race (% African American)	34.9	28.6	0.61
Body Surface Area (m^2^)	2.0 ± 0.3	2.0 ± 0.2	0.19
Tobacco Use (%)	65.9	66.7	0.95
Alcohol Use (%)	37.5	57.1	0.14
Hypertension (%)	95.3	85.7	0.18
Coronary Artery Disease (%)	72.1	47.6	0.06
Atrial Fibrillation (%)	30.2	14.3	0.17
Beta Blockers (%)	79.1	57.1	0.07
Calcium Channel Blockers (%)	32.6	23.8	0.47
ACE Inhibitors (%)	27.9	14.3	0.23
Angiotensin Receptor Blockers (%)	27.9	28.6	0.96
Mild Diastolic Dysfunction (%)	23.3	9.5	0.16^*^
Moderate Diastolic Dysfunction (%)	76.7	85.7	
Severe Diastolic Dysfunction (%)	0.0	4.8	

*Note*: Continuous data expressed as mean ± standard deviation. Categorical variables expressed as percentage

^*^ *p*-value calculated using Fisher’s Exact Test for a 2x3 contingency table

**Table 6:** Echocardiographic markers of vascular stiffness for diabetic subset

Variable	Group 1: Diabetic HFpEF (*n* = 43)	Group 2: Diabetic Diastolic Dysfunction (*n* = 21)	*p*-value
Arterial Stiffness (mmHg/mL/m^2^)	2.2 ± 0.6 - (14)	1.7 ± 0.6 - (9)	0.051
Arterial Elastance ± mmHg/mL)	1.9 ± 0.4 - (14)	1.7 ± 0.3 - (9)	0.098
Aortic Strain (%)	6.9 ± 3.3 - (43)	9.7 ± 5.6 - (9)	0.02
Aortic Distensibility (cm^2^dyne^−1^10^−3^)	1.8 ± 1.0 - (17)	3.5 ± 2.6 - (14)	0.02

*Note*: All data expressed as mean ± standard deviation. Group size is listed in parentheses

## Discussion

Our study found that in subjects with asymptomatic diastolic dysfunction, decreased aortic distensibility identified on TTE is associated with an increased risk of developing HFpEF compared to similar subjects with normal aortic distensibility on TTE. These findings extend to the diabetic population with asymptomatic diastolic dysfunction, in which both decreased aortic distensibility and aortic strain were shown to precede the development of HFpEF.

Our findings can be explained by the following pathophysiologic model, initially proposed by Massie in 2003: as the aorta becomes less elastic in the setting of arteriosclerosis, aging and hypertension, parallel structural changes of hypertrophy and fibrosis occur in the cardiac myocardium because of increased vascular load, leading to decreased myocardial compliance, impaired relaxation and left ventricular diastolic dysfunction. Over time, chronically elevated diastolic pressures in the left ventricle lead to the development of symptomatic heart failure [19]. Gillebert highlighted the concept of load-dependent diastolic dysfunction, a physiologic process by which afterload that crosses a particular threshold causes slow and incomplete myocardial relaxation, resulting in elevated filling pressures and ultimately congestive heart failure [20]. In 2011, Borlaug and Kass further refined the model by incorporating the concept of ventricular-arterial coupling into the pathophysiological development of HFpEF. In brief, the net interaction between ventricular and arterial stiffness impacts cardiac function and dictates the development of symptoms due to a greater dependence on systolic pressure for coronary flow and increased ischemia for a given drop in systolic blood pressure, which is exacerbated by exercise in patients with HFpEF [21]. In support, Ikonomidis et al. found that impaired left ventricular untwisting is associated with increased arterial stiffness, increased markers of collagen turnover and decreased coronary flow reserve in subjects with hypertensive heart disease, suggesting that impaired ventricular-arterial coupling precedes the development of HFpEF [22]. While brachial pulse pressure has historically been used as a crude surrogate for arterial stiffness, Naka et al. suggested in 2015 that the prognostic role of central aortic pressure, a potentially more robust marker of risk in heart failure, needs to be further investigated [23].

Chirinos et al. also demonstrated the concept of pulsatile load, which suggests that wave reflections that arise in peripheral arteries and return to the proximal aorta during mid-to-late systole are important contributors to left ventricular afterload. Their study demonstrated that increased arterial wave reflections during mid-to-late systole are associated with an increased risk for cardiovascular events and the development of heart failure symptoms [24]. Thus, it is possible that increased pulsatile load over time results in chronically elevated pressures in the ascending aorta, resulting in increased aortic stiffness and left ventricular afterload, and ultimately the development of HFpEF by inducing structural and functional changes in the left ventricular myocardium as described in the literature above.

When measuring proximal aortic stiffness, it is important to account for the dynamic effect of pulsatile load on the compliance of the aorta [25]. The equation used to measure proximal aortic distensibility includes pulse pressure as well as aortic strain; therefore, it accounts for a more physiologic and continuous measurement of aortic stiffness [12]. Even though other reliable non-invasive methods such as brachial-ankle pulse wave velocities take into account the peripheral vascular bed [26], the advantage of measuring proximal aortic stiffness from TTE is that it can still be assessed at the same time diastolic dysfunction is discovered in an individual subject without the need for additional testing.

Increased arterial stiffness has previously been correlated with diastolic dysfunction [27–29]. Namba et al. showed evidence in a cross-sectional study that cardio-ankle vascular index was independently associated with LV diastolic dysfunction in subjects with cardiovascular disease [27]. The study findings reported by Seeland et al. and Alba et al. support the significant associations between pathologic pulse wave velocities and the prevalence of diastolic dysfunction in women [28, 29]. Therefore, the increased correlation between arterial stiffness and asymptomatic diastolic dysfunction in subjects may also account for the lack of statistical significance between the two groups, each with diastolic dysfunction in our study. However, the statistically significant difference in aortic distensibility found between the two groups in our study suggests that even though increased peripheral arterial stiffness is associated with asymptomatic diastolic dysfunction, the additional contribution of increased proximal aortic stiffness may be required for the progression from asymptomatic diastolic dysfunction to HFpEF. In other words, it may be that the increased stiffness of the ascending aorta rather than the peripheral vasculature is the pathologic mechanism which drives the progression from diastolic dysfunction to HFpEF. Prospective studies are needed to further investigate the contribution of proximal aortic stiffness to the development of HFpEF.

### Strengths

The strengths of our analysis included the longitudinal nature of the study, which allowed us to investigate whether increased vascular stiffness precedes the development of HFpEF in patients with asymptomatic diastolic dysfunction. In addition, our matched cohort design allowed us to control for confounding variables including age, race and gender, all of which are known to be associated with vascular stiffness [28, 30, 31]. Subjects were also matched for BSA because BSA is a key determinant of aortic root and arch dimension [32]. In addition, matching allowed us to use a smaller sample size as compared to an unmatched study. Furthermore, our calculated values for aortic distensibility and aortic strain were similar to those published in previous literature, particularly for hypertensive and diabetic patients [18].

### Limitations

The number of subjects and retrospective design are important limitations of the study and any associations discussed should be considered in this context. Our sample size was further limited due to missing data in the analyzed TTE reports. For instance, 48/77 subjects in Group 1 and 28/77 subjects in Group 2 did not have blood pressure recordings at the time of their echocardiogram, rendering it impossible to calculate all the indices of vascular stiffness for these subjects. We did counter this limitation by including aortic strain as part of our analysis, so that at minimum we could assess proximal aortic stiffness in the absence of blood pressure recordings in all subjects. Despite this, arterial stiffness, arterial elastance and aortic strain were not significantly correlated with the development of HFpEF. A larger sample size may be needed to demonstrate statistically significant results, particularly in the diabetic sub-group.

Since 2003, the guidelines for assessing and grading diastolic dysfunction have been updated twice, most recently in 2016 [33]. Therefore, it is possible that some echocardiograms interpreted as having diastolic dysfunction or that some studies that were assumed to have normal diastolic function in 2003 would no longer receive the same classification under the new guidelines. However, incorporating peak E and A-wave velocities and E/A ratio into the determination of diastolic function has not changed since 2002.

Subjects were not matched for co-morbidities commonly seen in HFpEF such as hypertension, DM and CKD because we wanted to confirm the validity of our data set by reproducing the previously demonstrated association between the development of HFpEF and these co-morbidities. These covariates were adjusted for in the multiple logistic regression analysis to eliminate the effect of these variables on HFpEF development.

## Conclusions

This study confirmed the previously demonstrated association between the development of HFpEF and the presence of hypertension, DM, CAD, CKD and AF [5, 11–13]. However, the presence of decreased aortic distensibility on TTE in patients with diastolic dysfunction prior to the development of HFpEF is a novel finding.

In summary, these data support the conclusion that incorporating the simple measurements and calculations of proximal aortic stiffness, including distensibility and strain, when diastolic dysfunction is first identified on TTE may help to identify higher risk patients prior to the development of HFpEF. Larger prospective studies are needed to further investigate this relationship and to determine whether early interventions to control blood pressure and diabetes can alter the outcome in these patients.

## Abbreviations

AF: Atrial fibrillation
ANOVA: Analysis of variance
ASE: American Society of Echocardiography
AUC: Area under curve
BP: Blood pressure
BSA: Body surface area
CAD: Coronary artery disease
CHF: Congestive heart failure
CKD: Chronic kidney disease
d.f.: Degrees of freedom
DM: Diabetes mellitus
DWS: Diastolic wall strain
EACVI: European Association of Cardiovascular Imaging
EF: Ejection fraction
EHR: Electronic health record
ESP: End systolic pressure
FMLH: Froedtert Memorial Lutheran Hospital
FS: Fractional shortening
GFR: Glomerular filtration rate
HFpEF: Heart failure with preserved ejection fraction
ICD-9: International Classification of Diseases, 9^th^ Edition
LV: Left ventricular
LVEDV: Left ventricular end diastolic volume
LVEF: Left ventricular ejection fraction
LVESV: Left ventricular end systolic volume
LVIDd: Left ventricular internal diameter at end-diastole
LVIDs: Left ventricular internal diameter at end-systole
LVPWd: Left ventricular posterior wall thickness at end-diastole
LVPWs: Left ventricular posterior wall thickness at end-systole
MRI: Magnetic resonance imaging
NT-proBNP: N-terminal pro-hormone brain natriuretic peptide
PP: Pulse pressure
ROC: Receiver operating characteristic
RWT: Relative wall thickness
SAS: Statistical Analysis System
SV: Stroke volume
SV: Stroke volume
SVI: Stroke volume index
TR: Triscuspid regurgitant
TTE: Transthoracic echocardiogram
